# *Pimpinella anisum* L. Essential Oil a Valuable Antibacterial and Antifungal Alternative

**DOI:** 10.3390/plants12132428

**Published:** 2023-06-23

**Authors:** Eugenia Dumitrescu, Florin Muselin, Emil Tîrziu, Mihai Folescu, Carmen S. Dumitrescu, Dora M. Orboi, Romeo T. Cristina

**Affiliations:** 1 Faculty of Veterinary Medicine, University of Life Sciences “King Mihai I” from Timisoara, Calea Aradului 119, 300645 Timisoara, Romaniaflorinmuselin@usab-tm.ro (F.M.); emiltirziu@usab-tm.ro (E.T.); mikefolescu@gmail.com (M.F.); 2 Department Systems in Agriculture, Faculty of Management, University of Life Sciences “King Mihai I” from Timisoara, 300645 Timisoara, Romania

**Keywords:** *Pimpinella anisum*, essential oils, composition, antimicrobial, *Staphylococcus aureus*, *Escherichia coli*, *Pseudomonas aeruginosa*, *Streptomyces pyogenes*, *Candida albicans*

## Abstract

Anise (*Pimpinella anisum* L.*)* essential oils are intensely investigated worldwide for the beneficial properties, due to the specific bioactive compound’s structure. (1) Background: This study characterized the structure of the *Pimpinella anisum* essential oil and evaluated its antimicrobial properties. (2) Methods: An evaluation of the antibacterial and antifungal activity targeted strains of *Escherichia coli* (ATCC 25922), *Pseudomonas aeruginosa* (ATCC 27853), *Staphylococcus aureus* (ATCC 25923), *Streptococcus pyogenes* (ATCC 19615), and levure *Candida albicans* (ATCC 10231). Gas chromatography coupled with mass spectrometry (GC/MS) was used for structure identification, and the optical density mass loss was applied for the analysis of different dilutions of aniseed essential oils antimicrobial activity. (3) Results: A total of 13 compounds were identified, of which trans-anethole was in the highest proportion (72.49%), followed by limonene (10.01%), anisole (5%), and α-pinene (3.26%). The results obtained and statistically analyzed, utilizing one-way ANOVA with Bonferroni’s multiple comparison test, indicated the antimicrobial activity (*p* < 0.001) of anise essential oil. (4) Conclusion: Anise essential oil is a promising phyto-remedy with important antimicrobial activity against both Gram-positive and Gram-negative pathogens. Inhibition high percentages were found for the *p. aeruginosa* and *S. aureus* strains, but also excellent antifungal activity against *C. albicans* was ascertained.

## 1. Introduction

The history of aromatic anise seeds use begins as early as 5000 years ago, and it is very difficult to determine the first recorded use of anise. Some investigators believe that anise originates from Ancient Egypt, while others attribute it to Greece or even Persia (the current territory of Iran) [1].

Anise is part of the *Apiaceae* family and is an aromatic, herbaceous, annual, heliophilous plant that prefers a warm climate. Cultivated since ancient times in Asia Minor, its use later spread to Europe and other continents [2,3]. The plant has few leaves, with small and white flowers arranged in the form of an umbrella. The fruits are small and green, ripening from the end of August to the end of September. They are small diachenes, ovoid, with hard-to-separate halves, with five slightly prominent ribs, lighter in color [2].

The fruits contain numerous substances and essential oils, namely anethole, present in large amounts, as well as anisaldehyde, anisic acid, and eugenol. Anise fruits also contain other fatty, proteinaceous, carbohydrate substances; mucilage; anethole; coumarins; tocopherol; and polyphenol carboxylic acids. Some substances that demonstrate an estrogenic effect are also mentioned in relevant works [4].

The seeds contain at most 6% essential oil, of which up to 90% is anethole (an organic compound commonly used as a natural flavor and main component of essential oil extracted from plants) and gives the plant its typical aroma. Coumarins, flavonoids, and sterols can be found in the composition of the seeds [5].

The presence of eugenol, trans-anethole, methyl-chavicol, estragole, coumarin, anisaldehyde, estrols, terpene hydrocarbons, polyenes, scopoletin, and polyacetylenes as the main compounds of aniseed essential oil were illustrated in numerous studies [6,7].

The GC and GC-MS analysis of *P. anisum* fruits essential oils revealed the presence of trans-anethole (93.9%) and estragole (2.4%). In concentrations greater than 0.06%, α-cuparene, β-bisabolene (E)-methyl-eugenol, α-himachalene, p-anisaldehyde, and cis-anethole were ascertained [1,8,9].

The chemical components of the anise extract examined by GC-MS after supercritical extraction using CO2 revealed that anethole (~90%), γ-hymacalene (2–4%), p-anisaldehyde (<1%), methyl-chavicol (0.9–1.5%), cis-pseudoisoeugenyl, 2-methyl-butyrate, and trans-pseudoisoeugenyl 2-methyl-butyrate (~1.3%) were the main compounds identified [7,10,11,12]. One of the phenolic glycosides of the *Umbelliferae* family (4-(p-d-glucopyranosyloxy) benzoic acid) was also found in anise [13,14].

This plant has several pharmacological properties, including the following:

*a.*  *Antibacterial, antifungal action*

Authors studied the antibacterial activity of *Pimpinella anisum* extracts made in methanol, acetone, petroleum ether, and water. These extracts were tested on four pathogenic bacteria (e.g., *Staphylococcus pyogenes, Streptococcus aureus*, *Klebsiella pneumoniae,* and *Escherichia coli*) using the disc diffusimetric method [15]. Only the methanolic and aqueous extracts showed relevant antibacterial activity against all tested bacteria and that the aqueous extract appeared to be more effective when compared to the methanolic extract. The acetone and petroleum ether extracts were not shown to inhibit the growth of the tested pathogenic bacteria [16].

The effects of aqueous and ethanolic extracts of anise were investigated on 10 bacterial species as well as on *Candida albicans* by utilizing the disc diffusimetric method. In this study, the ethanolic extract showed significant inhibitory activity against all bacteria tested but was not found to be effective against *Candida albicans*. However, the antimicrobial effect of the aqueous extract was not illustrated against Gram-negatives *Escherichia coli* and *Pseudomonas aeruginosa*. Furthermore, *Candida albicans* was sensitive to the effect of the aqueous extract [17]. Alcoholic extracts obtained from *P. anisum* seeds validated the antibacterial activity against *Micrococcus luteus* and *Mycobacterium smegmatis* [18,19].

Some authors emphasized that, besides the antibacterial activity, anise essential oil also exhibits significant antifungal activity. The authors claimed that this is due to the anethole in the composition of the oil [20]. In their research, the authors examined the antifungal activity of the essential oil obtained from anise seeds and of the aqueous extract on four species of dermatophytes and seven species of yeasts via the cylinder diffusion method and broth dilution method. The authors illustrated the fact that the aqueous extract of anise displayed antifungal activity against *Candida* (species *albicans, krusei, parapsilosis*, *pseudotropicalis,* and *tropicalis*), the largest inhibition being observed in *C. albicans* [20].

An inhibitory effect was also demonstrated on dermatophyte species (*Trichophyton rubrum*, *T. mentagrophytes*, *Microsporum canis,* and *M. gypseum*), the most pronounced zone of inhibition detected being for *T. mentagrophytes* [21].

There are also some studies that demonstrate the antibacterial effectiveness of other essential oils, such as tea tree essential oil on *E. coli*, *S. typhi*, *C. koseri*, and *S. aureus* but also juniper essential oil on strains of *Staphylococcus aureus* [22,23].

*b.*  *Anticonvulsant action*

The anticonvulsant effects of the *Pimpinella anisum* essential oil present in the fruits were analyzed on seizures induced using pentylenetetrazole (PTZ) and maximal electroshock (MES) in male mice. The authors showed that *P. anisum* boosted the threshold of clonic seizures caused by the i.v. introduction of PTZ and also blocked tonic convulsions that appear upon the i.p. injection by PTZ. Furthermore, *P. anisum* possessed anticonvulsant activity against tonic seizures induced by maximal electroshocks [24].

*c.*  *Action on the digestive system*

The effect of aqueous anise extract on gastric ulceration in rats was studied, the acute gastric ulceration being induced by various harmful chemicals and the administration of indomethacin. The results of the study showed that anise significantly inhibited the damage to the gastric mucosa induced by indomethacin and necrotizing agents. The antiulcer effect was corroborated both by the authors and histologically [25].

*d.*  *Analgesic and anti-inflammatory action*

There are authors who demonstrated that the essential oil of *Pimpinella anisum* administered to mice presented a significant analgesic effect similar to that of morphine and aspirin [26]. Research has shown that anise essential oil has an anti-inflammatory effect as strong as indomethacin and has an analgesic effect similar to that of 10 mg/kg.bw. of morphine and 100 mg/kg.bw. of aspirin after 30 min [27,28].

*e.*  *Antioxidant action*

In a study, the antioxidant properties of ethanolic and aqueous extracts of anise were studied. The antioxidant properties were assessed employing different antioxidant assays and were contrasted with synthetic antioxidants, for instance, butylated hydroxytoluene (BHT), butylated hydroxyanisole (BHA), and tocopherol. Both studied extracts demonstrated strong antioxidant activity by scavenging DPPH radicals and superoxide anions, absorption of hydrogen peroxides, as well as capabilities to perform metal chelation compared to BHT, BHA, and α-tocopherol. The authors concluded that, of the two extracts studied, it seemed that the aqueous extract had a higher antioxidant capacity when compared to the ethanolic extract [17].

*f.*  *Acaricidal activity*

The authors also studied the acaricidal activity of p-anisaldehyde derived from aniseed essential oil against house dust mites *Dermatophagoides pteronyssinus* and *Dermatophagoides farina*. The results of this study showed that benzyl-benzoate and p-anisaldehyde were the compounds that had the most significant effect on these dermatophagoides. Therefore, p-anisaldehyde can be used for the selective control of house dust mites [29].

*g.*  *Antitumor activity*

Researchers investigated the cytotoxic effect of both the extract and essential oil of *P*. *anisum* on the AGS (gastric cancer) cell line. The results illustrated the cytotoxic effect of alcoholic extracts and essential oils on gastric cancer cells at concentrations of 15 to 480 μg/mL but did not demonstrate any significant effect on fibroblast cells as normal cells. The inhibitory effect on the growth of AGS cells was much greater in the case of the methanolic extract compared to the ethanolic extract [30].

Therefore, *P*. *anisum* may present a natural source of compounds with antiproliferative properties. In tumor growth, blood vessel formation (angiogenesis) is a necessary stage, supplying oxygen and nutrients to tumoral cells. This process can contribute to tumor progression, invasion, and metastasis. It is commonly used as an indicator of tumor prognosis. Therefore, tumor angiogenesis has been determined to be of high clinical relevance. The authors of this study evaluated the effects of extracts and essential oil on the process of angiogenesis [30].

*h.*  *Neural activity*

Few studies indicate the possible effects of *Pimpinella anisum* essential oil on neural activity. The plant and, especially, the essential oils have been used in the treatment of certain neurological diseases, including epilepsy and seizures.

There is research attempting to evaluate the possible antidepressant and anxiolytic effects of anise oil and its ability to attenuate in vivo*,* Pb-induced cognitive deficits in rats exposed to lead during gestation and lactation. The result demonstrated that exposure to lead during intrauterine development causes problems with body development and brain weight development and increases the level of anxiety, depression, and locomotor hyperactivity in rats exposed to lead compared to the control rats. The authors claimed that administration of *Pimpinella anisum* essential oil to lead-exposed rats led to a reduction in anxiety, depression, and locomotor hyperactivity. The results of the study demonstrated that lead exposure during intrauterine development induced a significant disruption of emotional reactivity that can be improved by treatment with *Pimpinella anisum* essential oil [31].

Anise essential oil is used as an expectorant, carminative, and in cough mixtures, especially in pediatrics, and important phenylpropanoids such as trans-anethole and estragole have a stabilizing influence on the autonomic nervous system. The aim of the present research was to evaluate the chemical composition and antimicrobial activity of a commercially procured anise essential oil.

## 2. Results

### 2.1. Phytochemical Compounds Identification

The identification of the compounds from the anise essential oil was carried out using the Agilent gas chromatograph *(Agilent Scientific, Santa Clara CA, USA)* paired with the mass spectrometer, revealing 13 different compounds. 

As the most important compounds among those identified, α-pinene (3.263%), anisole (5.003%), limonene (10.011%), and propenyl-anisole (72.499%) peaks were ascertained for the sampled oils, representing 90.776% from the total compound structure.

Table 1 presents the compound structure identified in the studied essential oil.

The chromatogram peaks are presented in the attached Supplementary Figure S1.

### 2.2. Antimicrobial Activity

**Figure 1:**
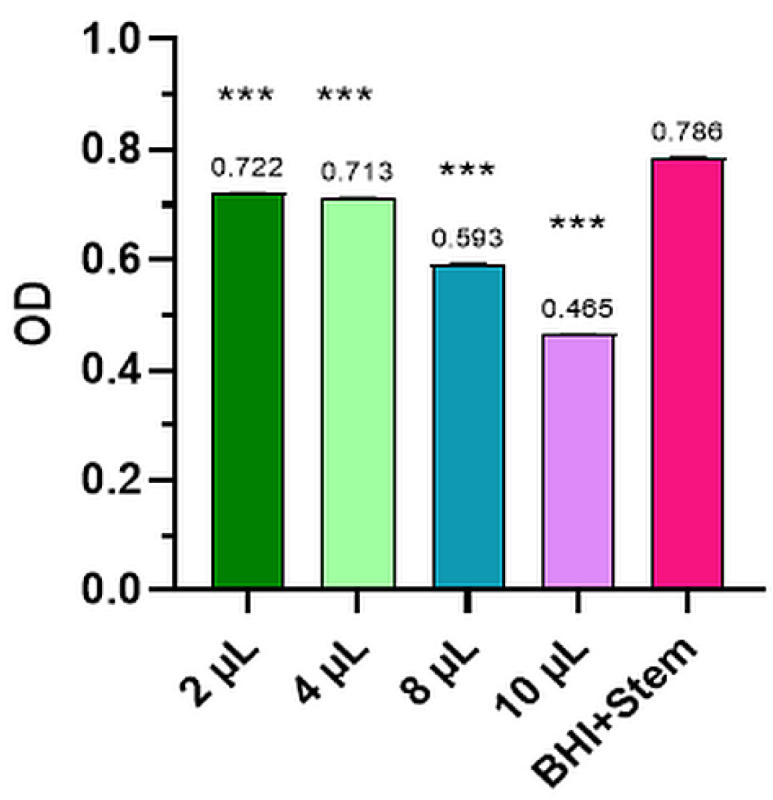
*Streptococcus pyogenes*—mean values of the three determinations for the Control and the four dilutions tested (where *** means *p* < 0.001).

**Figure 2:**
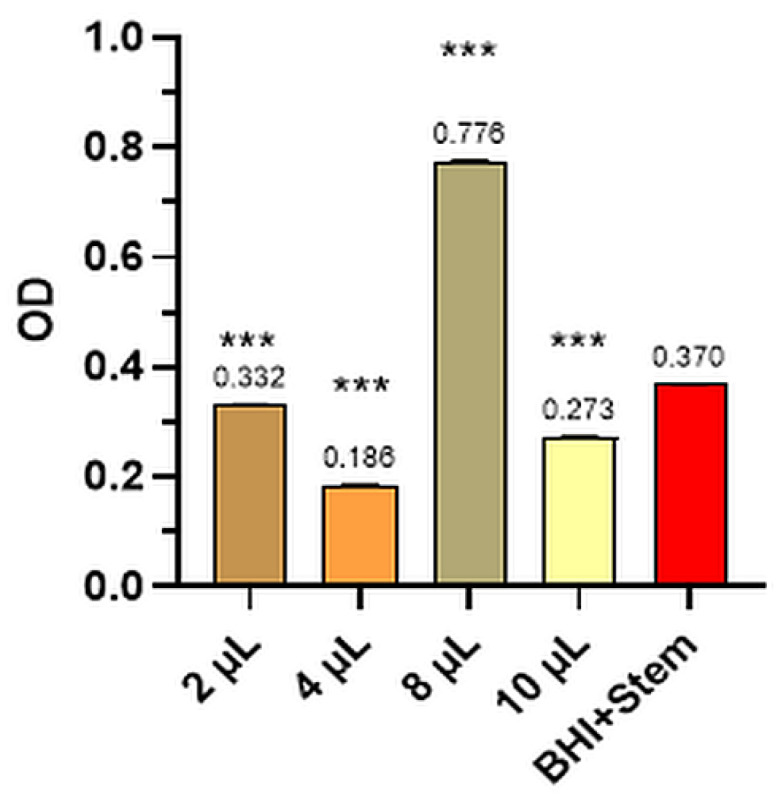
*Pseudomonas aeruginosa*—mean values of the three determinations for the Control and the four dilutions tested (where *** means *p* < 0.001).

**Figure 3:**
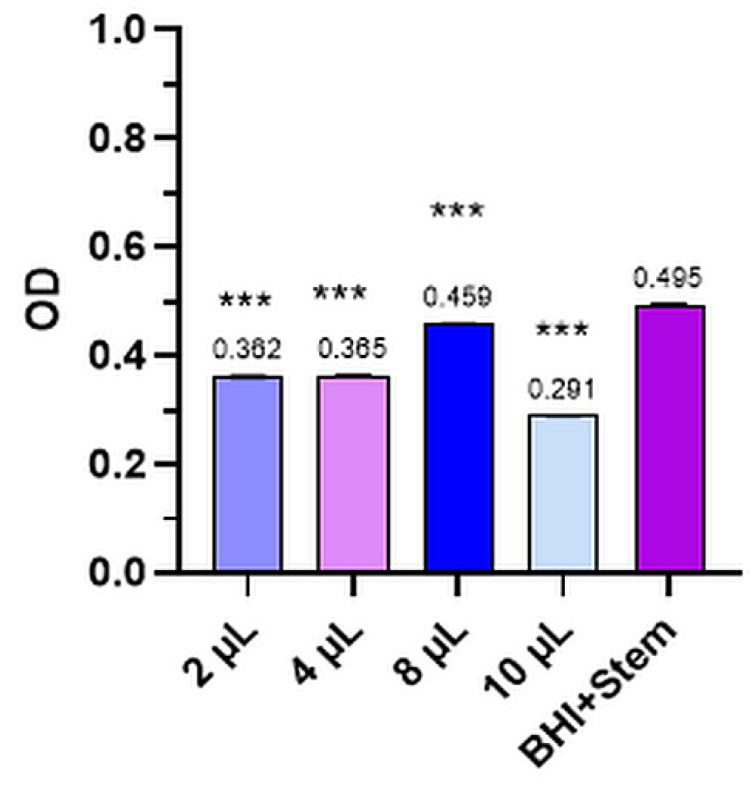
*Staphylococcus aureus*—mean values of the three determinations for the Control and the four tested dilutions (where *** means *p* < 0.001).

**Figure 4:**
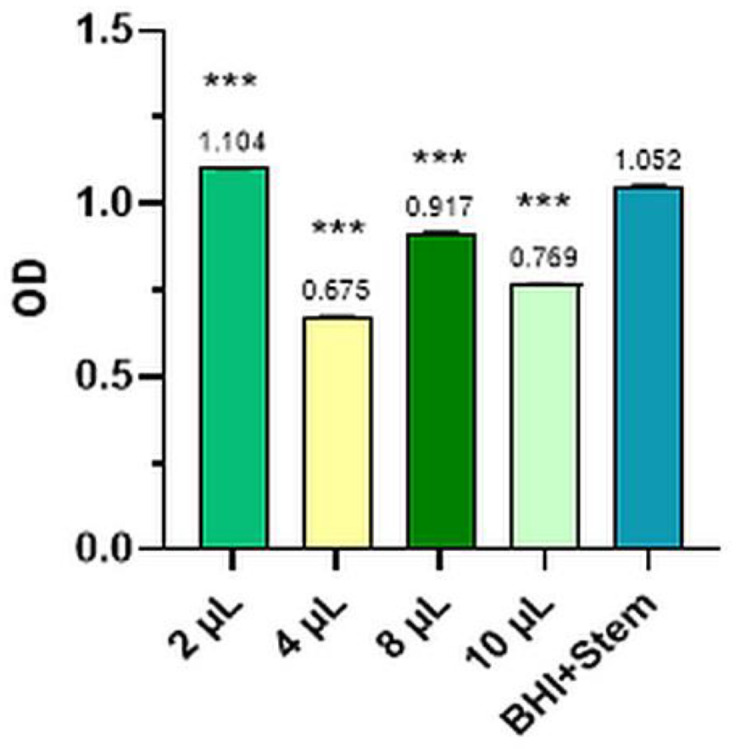
*Escherichia coli*—mean values of the three determinations for the Control and the four tested dilutions (where *** means *p* < 0.001).

**Figure 5:**
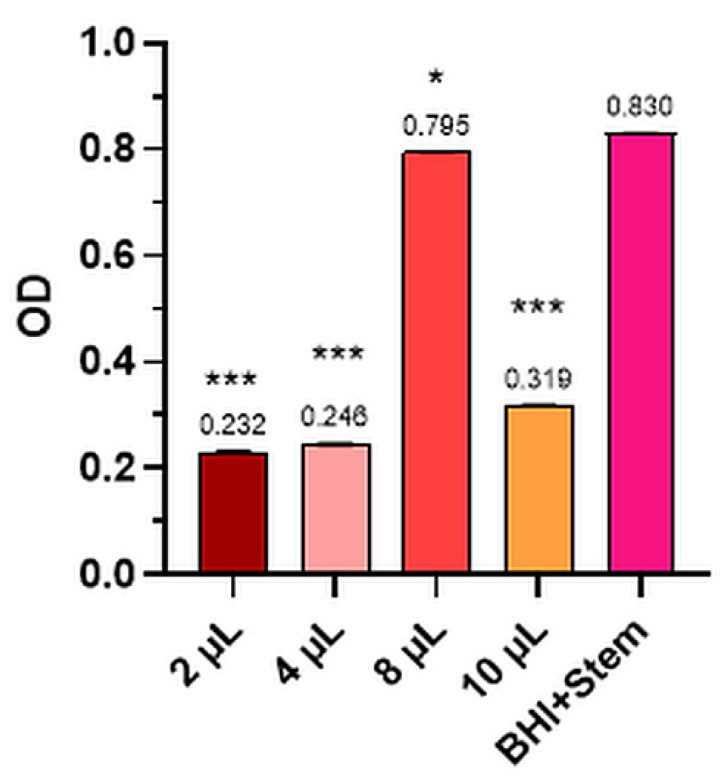
*Candida albicans*—mean values of the three determinations for the Control and the four tested dilutions (where *** means *p* < 0.001 and * means *p* < 0.05).

The mean values of the three determinations for the Control and the four dilutions tested for bacterial strains and levure and the comparative optical densities (OD) for anisum essential oil tested for the inhibition of *S. pyogenes*, *P. aeruginosa*, *S. aureus*, *E. coli*, and *C. albicans* are presented in Figure 1, Figure 2, Figure 3, Figure 4, Figure 5 and Table 2.

#### 2.2.1. *Streptococcus Pyogenes*

All anise essential oils at dilutions of 2, 4, 8, and 10 μL hindered the development of *Streptococcus pyogenes* (ATCC 19615) to different degrees. The evaluation of the antimicrobial activity of anise essential oils, performed using optical density mass loss reveal in Figure 1 the optical density (OD) of the anise essential oil and the Control.

Increasing the volume of anise oil to 10 μL led to a decline in bacterial cell density from 0.786 ± 0.005 (Control) to 0.465 ±0.003 (the 10 μL dilution), a notable value when compared to the Control sample, also covered statistically (*p* < 0.001). In the Control group, the optical activity was thought to present a potency of 100% and, in the case of the oil currently tested, inhibition of the development of *Streptococcus pyogenes* strains of 40.86% was observed for the 10 μL dilution.

#### 2.2.2. *Pseudomonas Aeruginosa*

Figure 2 shows the optical density (OD) of the anise essential oil and the Control for the *Pseudomonas aeruginosa* bacteria strain.

Anise oil dilutions impeded the development of the *Pseudomonas aeruginosa* (ATCC 27853) strain. Remarkably, the highest inhibition percentage was obtained at dilutions of 8 μL (109.84%) and, respectively, 4 μL (49.59) of the essential oil, compared to the Control, leading to a decline in bacterial cell density from OD = 0.370 ± 0.008 (the Control) to OD = 0.186 ± 0.001 (dilution of 4 μL), and close to the value of 10 μL dilution (OD = 0.273 ± 0.001), results statistically confirmed (*p* < 0.001).

#### 2.2.3. *Staphylococcus Aureus*

Figure 3 illustrates the optical density of the anise essential oil as well as the Control for the *Staphylococcus aureus* standard strain.

In the case of the *Staphylococcus aureus* (ATCC 25923) strain, anise essential oil inhibited its growth compared to the Control (OD = 0.495 ± 0.006) by a 41.18% inhibition rate, at a dilution of 10 µL and (OD = 0.291 ± 0.005) (*p* < 0.001). Thus, we can say that the highest volume also had the highest inhibition rate in the case of *Staphylococcus aureus* strain.

#### 2.2.4. *Escherichia Coli*

The optical density of anise essential oil in the case of *Escherichia coli* is shown in Figure 4.

In the case of *Escherichia coli* (ATCC 25922), the percentage of inhibition produced by anise essential oil was 35.83% at a dilution of 4 µL (OD = 0.675 ± 0.009) and 26.90% at a dilution of 10 µL (OD = 0.769 ± 0.008) (*p* < 0.001).

#### 2.2.5. *Candida Albicans*

The optical density of anise essential oil in the case of levure *Candida albicans* is shown in Figure 5.

In the case of *Candida albicans* (ATCC 10231), the percentage of inhibition produced by anise oil was 72.03% compared to the Control (OD = 0.830 ± 0.007) for 2 µL dilution (OD = 0.232 ± 0.007) and 61.60% compared to the Control for a dilution of 10 µL (0.319 ± 0.008). In the case of the levure *Candida albicans*, we can say that *Pimpinella anisum* essential oil was much more effective at the lowest dose used.

In Table 2, the comparative optical densities (OD) for anisum essential oil tested for the inhibition of *S. pyogenes, P. aeruginosa, S. aureus, E. coli,* and *C. albicans* are presented.

Analyzing the inhibition rate percentage (IRP), we perceived that in the case of Gram-negative strains (*Pseudomonas aeruginosa* and *Escherichia coli* in our case) and of the *Candida albicans* levure, the IRP was greater in the case of the lower dilutions in all cases, with 10 µL dilution generating inferior results.

## 3. Discussion

Following the chromatographic analysis of anise essential oil, 13 compounds were identified, the major ones being propenyl-anisole (syn. trans-anethole) in proportion to 72.49%, limonene 10.01%, anisole 5%, and α-pinene (3.263%). These results are consistent with those of other authors of the domain, who identified trans-anethole, a phytoestrogen, as the major compound in *Pimpinella anisum* essential oil [32,33,34,35,36].

Moreover, in a study conducted by Orav et al. [7], the main component of *Pimpinella anisum L*. fruits essential oil obtained from various geographical areas of Europe was trans-anethole. Chemical characterization of essential oils is very important for understanding their biological properties. Plants are known to produce many secondary metabolites, of which most have distinctive pharmacological actions, including phenolic acids, flavonoids, and tannins [37].

Pure essential oils are mixtures of over 200 components, normally mixtures of terpenes or phenylpropane derivatives. They can be largely categorized into two groups:

- Volatile fraction: constituting 90–95% of the oil and containing monoterpene and sesquiterpene hydrocarbons with known derivatives (i.e., oxygenated, aliphatic aldehydes, alcohols, and esters fractions).
- The non-volatile residues: comprising only 1–10% of the oil, containing hydrocarbons, fatty acids, sterols, carotenoids, wax, and flavonoids [37].

Several factors influence the quantity and quality of the extract, especially the composition of the soil, the plant organ, the phase of the vegetative cycle, and the climate, and this is why it can be affirmed that composition is geographically related [38].

The presented results are consistent with those obtained by researchers [18], who found a high inhibitory effect of anise essential oil on relevant bacteria (*E. coli, E. faecalis, M. luteus, S. typhi,* and *S. aureus*) [18].

Al-Bayati [19] also confirms the strong inhibitory action of *Pimpinella anisum* essential oil against bacteria and fungi, such as *Staphylococcus aureus*, *Escherichia coli*, and *Candida albicans*. In the case of essential oils, antagonistic, additive, and synergistic effects were ascertained. When these components’ effect equals the summation of the different effects, the additive effect will emerge. In this study, carried out comparatively on the effectiveness of *Pimpinella anisum* and *Thymus vulgaris* essential oils against *P. aeruginosa*, the authors demonstrated that they were inactive at the highest concentration used (500.0 μg/mL) when applied individually, while their association (1:1) impeded the growth of the pathogen *P. aeruginosa* [19].

Synergism represents an overall activity greater than the total individual effects taken separately. Conversely, the antagonistic effect is recorded if the activity of the components in combination is lower, when compared to their effect applied separately [37]. 

There has been an increase in studies performed on the plant essential oils mechanisms. However, compared to many studies on the characteristics of essential oils (EOs) and their components and the number of studies investigating the specific target(s) of the antimicrobial action of EOs and their components, a significant field of study remains. The antimicrobial action of EOs is related to one of the most important characteristics of them, respectively, their hydrophobicity leading to increased cellular permeability and, consequently, leakage of cellular constituents. It is important to understand that a disrupted cellular structure can affect other cellular structures in a cascade type of activity [37].

There are authors who claim that, in addition to the antibacterial activity, anise essential oil also has noteworthy inhibitory activity against fungi, and the most active component found was anethole [39].

Kosalec et al. [21] demonstrated that anise essential oil has strong antifungal activity against dermatophytes and yeasts, the widest zone of inhibition appearing for *Candida parapsilosis*, followed by *Geotrichum spp*, *C. glabrata,* and *C. albicans*. [21].

The essential oils are complex mixtures with a great diversity of components and consequently their promising antimicrobial activity is related to their composition, configuration, amount, and possible interactions [40,41]. 

To date, various studies have been conducted on the essential oils and extracts of *Pimpinella anisum* in order to ascertain the chemical compounds and pharmacological properties of this plant. Various beneficial properties have been reported, including the antimicrobial, antifungal, antiviral, antioxidant, and insecticidal effects of anise [42].

The strains that most commonly develop resistance were used; therefore, the obtained results could be considered as significant. They open novel and noteworthy applications in phytotherapy, in the frame of the One Health concept which strongly suggests the use of essential oils as valuable phyto-resource alternatives in antibiotic resistance. The gathered information can be applied in designing new antimicrobial associations or therapeutical agents. To fully investigate the *Pimpinella anisum* characteristics, further in vivo models are necessary and will remain to be studied soon.

In our case, analyzing the inhibition rate percentage (IRP), we perceived that in the case of Gram-negative strains (*Pseudomonas aeruginosa* and *Escherichia coli*) and of the *Candida albicans* levure, the IRP was greater in the case of the lower dilutions, in all these cases the 10 µL dilution generating inferior results. 

In the case of the *P. aeruginosa* standard strain, the inhibition value revealed the anise essential oil’s great potency, confirming that the antimicrobial potential of anise essential oils varies between Gram-positive and Gram-negative bacteria, due probably to density variations in the bacterial cell wall. As we know, essential oils are in preponderance lipophilic, and this is why they are rapidly absorbed by Gram-positive bacteria due to their dense layer (especially of lipophilic phospholipids in their cell walls).

This is why all studies tackling this issue could be considered of help. We consider that this class of studies is performed to develop the associative features of phyto-therapeutic means with other structures such as chemotherapeutics, sulfonamides, or antibiotics with the aim of gathering new efficient associated phyto-structures especially where the efficacy of anti-infectious classes is compromised by the resistance phenomena. 

## 4. Materials and Methods

The 100% pure anise *Pimpinella anisum-Apiaceae (Umberlliferae)* essential oil conditioning (*Aromatics International*) used in the study (Batch no. ANS-103/09.20.2020) was purchased from a local herbal store. According to the product’s information sheet, the manufacturer extracted the essential oil by steam distillation from organically grown brown pods of anise seed flowers, with a yield of 2–4%. Organoleptically, the oil has a grassy, sweet aroma with a slightly spicy (licorice-like) smell; its color is pale yellow with a medium viscosity.

The chromatographic and microbiological analysis of anise essential oil was carried out within the Interdisciplinary Research Platform and the Toxicology—Pharmacology Research Laboratory within the Faculty of Veterinary Medicine Timișoara.

### 4.1. The GC/MS Compounds Analysis in P. anisum Essential Oil

The analysis of the anise essential oil sample taken in the study was carried out using the Agilent Technology 7820A gas chromatograph, coupled with the Agilent MSD 5975 mass spectrometer (Agilent Scientific, Santa Clara, CA, USA) and with a DB WAX capillary column (30 m × 250 pm × 0.25 µm). Helium was used with a mass flow rate of 1 mL × min. For the separation of compounds, the following oven program was used: at 40 °C for 1 min, at 5 °C for 1 min, and raised to 210 °C for 5 min.

The temperatures for the injector and ion source were 250 and 150 °C, respectively. The injection volume was 1 µL of each pure, solvent-free oil or mixture at a ratio of 1:20.

The volatile compounds were identified using the National Institute of Standards and Technology (NIST) spectrum library. This was conducted by comparing the mass spectra with the ones stored in the NIST 02, (Wiley 275 libraries). The percentage value of individual components was calculated based on GC peak areas without making use of any correction factors. 

### 4.2. Determination of Antimicrobial Activity of P. anisum Essential Oils

The antimicrobial activity was tested in triplicate. Bacterial strain suspensions in BHI were used as a positive Control. Over each bacterial suspension well, the essential oil of anise was used directly by adding in increasing amounts as 2, 4, 8, and 10 µL / well. Plates were covered and left overnight at 37 °C; then, the OD (optical density) was measured at 590 nm using a Bio-Rad PR 1100 Elisa reader (Bio-Rad Laboratories, Inc., Hercules, CA, USA).

The average values of determinations were expressed statistically by one-way ANOVA. The differences were considered significant when *p* < 0.05 or lower.

#### Broth Micro Dilution Method

Among the most basic antimicrobial susceptibility testing methods is the broth microdilution method. This procedure tests 2-fold dilutions of the antimicrobial agent being assessed in 96-well micro titer plates filled with a liquid growth medium. Each plate is inoculated with microbes prepared in the same medium after dilution of the standardized microbial suspension adapted to the McFarland scale of 0.5.

After inoculation, the 96-well microtiter plate is incubated under appropriate conditions according to the microorganism tested.

The Minimum Inhibitory Concentration (MIC) is the lowest concentration of antimicrobial agent that completely inhibits the growth of the organism in the microdilution wells. The broth microdilution method has been standardized by the Clinical and Laboratory Standards Institute (CLSI) for testing aerobic bacteria, yeasts, and filamentous fungi. The European Committee on Antimicrobial Susceptibility Testing (EUCAST) broth microdilution method is similar to that of CLSI, with modifications typically relating to some of the test parameters, such as inoculum preparation, inoculum size, and MIC reading. The reference strains (ATCC) for the in vitro testing used were *Staphylococcus aureus* (ATCC 25923), *Escherichia coli* (ATCC 25922), *Pseudomonas aeruginosa* (ATCC 27853), *Streptococcus pyogenes* (ATCC 19615), and *Candida albicans* (ATCC 10231), provided from the Microbiology Lab facility, within the ULS “King Mihai I” from Timisoara. 

All samples were run in triplicate. A fresh culture dilution of 10^−3^ with an inoculum equivalent to a 0.5 McFarland standard was prepared and used. The ATCC strains used were revitalized in Brain Heart Infusion (BHI) broth (Oxoid, CM1135) (Oxoid Ltd., Basingstoke, UK) overnight at 37 °C and then plated on BHI agar for 24 h at 37 °C. Dilution was performed to an optical density (OD) of 0.5 McFarland standard (1.5 × 108 CFU/mL) using BHI broth.

The resulting suspensions were assayed using a 96-well flat-bottom microdilution plate with a usable volume of 200 µL. Using a Calibra 852 digital multi-channel pipette (Socorex Isba S.A. Ecublens, Switzerland), 100 µL of suspension was placed in each well. 

### 4.3. Statistical Analysis

The obtained results were presented as an arithmetic mean and SEM (Standard Error of the Mean) and were subjected to statistical analysis using one-way ANOVA (analysis of variance) with Bonferroni’s multiple comparison test. Statistically significant differences were considered when *p* < 0.05 or lower. The GraphPad Prism 6.0 software *(GraphPad Software, San Diego, USA)* was utilized for the analysis.

## 5. Conclusions

A chromatographic analysis of anise essential oil confirmed the massive presence of polyphenolic compounds. The antimicrobial activity of *Pimpinella anisum* essential oil was mainly related to its phenolic content, with propenyl-anisum, limonene, and anisole being preponderant. To this aim, anise essential oil had certain antimicrobial activity against both the Gram-positive and Gram-negative pathogens tested. The highest percentages of inhibition were found in order on the *Pseudomonas aeruginosa* (109.84%) and *Staphylococcus aureus* (41.21%) strains but also with excellent antifungal activity against *Candida albicans* (72.04%).

## Figures and Tables

**Table 1:** Compounds identified in anise essential oil.

Compound	R. Time	%
α-pinene	6.382	3.263
3-carene	9.847	1.327
α-phellandrene	10.309	1.822
Limonene	11.292	10.011
β-terpinen	11.548	2.511
1-pentanone, 1-(4-methylphenyl)	13.259	0.179
Linalool	20.840	1.962
Anisole	23.697	5.003
Propenyl-anisole	27.360	72.499
Anisaldehyde	31.552	0.326
p-(pentyloxy) acetophenone	34.046	0.089
Thymol	34.583	0.580
1-(3-methyl-2-butenoxy)-4-(1-propenyl) benzene	36.192	0.429

**Table 2:** The comparative optical densities for anisum essential oil tested, SEM values, and the inhibition percentage for *S. pyogenes*, *P. aeruginosa*, *S. aureus*, *E. coli,* and *C. albicans standard strains*.

*Streptococcus pyogenes*	**Concentration** **/Replica**	**I**	**II**	**III**	**x** **¯**	**SEM**	**Inhibition** **(%)**
	BHI+strain	0.786	0.787	0.786	0.7864	0.0005	100.00
	10 µL	0.465	0.466	0.464	0.465	0.0003	40.86%
	8 µL	0.593	0.591	0.594	0.5928	0.0005	24.61%
	4 µL	0.714	0.712	0.714	0.7132	0.0007	9.3%
	2 µL	0.722	0.724	0.721	0.7218	0.0006	8.21%
*Pseudomonas aeruginosa*	BHI+strain	0.368	0.370	0.371	0.3697	0.0008	100.00
	10 µL	0.274	0.268	0.276	0.2726	0.0019	26.28%
	8 µL	0.777	0.775	0.778	0.776	0.0008	109.84
	4 µL	0.184	0.189	0.186	0.1864	0.0010	49.59
	2 µL	0.331	0.332	0.334	0.3324	0.0008	10.11
*Staphylococcus aureus*	BHI+strain	0.496	0.495	0.495	0.4954	0.0006	100.00
	10 µL	0.292	0.289	0.291	0.2906	0.0009	41.18
	8 µL	0.459	0.458	0.461	0.4594	0.0006	7.26
	4 µL	0.366	0.365	0.363	0.3646	0.0010	26.44
	2 µL	0.362	0.360	0.366	0.3626	0.0013	26.71
*Escherichia coli*	BHI+strain	1.053	1.050	1.052	1.052	0.0008	100.00
	10 µL	0.769	0.767	0.771	0.769	0.0008	26.90
	8 µL	0.918	0.919	0.916	0.917	0.0010	12.77
	4 µL	0.678	0.674	0.673	0.675	0.0009	35.80
	2 µL	1.106	1.101	1.105	1.104	0.0009	4.92
*Candida albicans*	BHI+strain	0.831	0.832	0.828	0.8304	0.0007	100.00
	10 µL	0.319	0.317	0.320	0.3186	0.0008	61.60
	8 µL	0.796	0.795	0.795	0.7953	0.0007	4.23
	4 µL	0.244	0.244	0.247	0.245	0.0027	70.42
	2 µL	0.234	0.233	0.230	0.2323	0.0007	72.03

Where BHI = Brain Heart Infusion; x¯ = Arithmetic mean; SEM = Standard Error of the Mean.

## Data Availability

All data are included in the present manuscript.

## Supplementary Materials

The following supporting information can be downloaded at: https://www.mdpi.com/article/10.3390/plants12132428/s1, Figure S1: GC-MS analysis - The peaks of the compounds from *Pimpinella anisum* essential oil.
