# Supplementary material for: Pimpinella anisum L. Essential Oil a Valuable Antibacterial and Antifungal Alternative
Source: Plants (Basel). 2023 Jun 23;12(13):2428. doi: 10.3390/plants12132428 (PMC10347090; doi:10.3390/plants12132428)

Supplementary material

Supplementary Figure S1.

GC-MS analysis - The peaks of the compounds from *Pimpinella anisum* essential oil

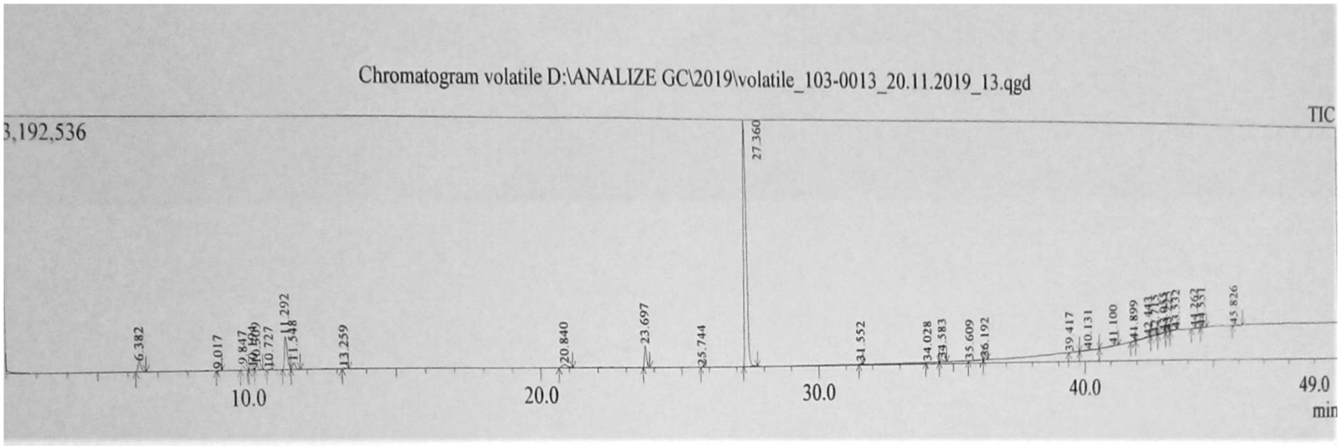

Supplement: Supplementary file 1 [file plants-12-02428-s001.zip › plants-2410165-supplementary.pdf]
